# Supplementary material for: An Intracellular Epitope of ASFV CD2v Protein Elicits Humoral and Cellular Immune Responses
Source: Animals (Basel). 2023 Jun 12;13(12):1967. doi: 10.3390/ani13121967 (PMC10295607; doi:10.3390/ani13121967)
Supplement: Supplementary file 1 [file animals-13-01967-s001.zip › Supplementary Tables.pdf]

Table S1. Primers for the eukaryotic expression of recombinant protein CD2v

Peptides	Sequences (5’-3’)
The full-length CD2v-F	gatcggatccggATGATCATCCTGATCTTCC
The full-length CD2v-R	gatcaagcttttaGATGATGCGATCGACG
The intracellular domain of CD2v-F	gatcggatccggtctttacgaaaaagaaaaaaacat
The intracellular domain of CD2v-R	gatctctagattaaataattctatctacgtgaataagcgaaatattttggg

Table S2. Sequences of primers to amplify the variable region of heavy and light chains of monoclonal antibodies

Peptides	Sequences (5’-3’)
VH-F	asrtcmagctgcaggagtctgg
VH-R	tgaggagacggtgaccgtggtcccttggccccag
VL-F	gacattgagctcacccagtctcca
VL-R	gttagatctcgagcttggtccc

Table S3. The corresponding nucleotide sequences of the peptides of CD2v

Peptides	Sequences (5’-3’)
CD2v (262-271aa)	ATCCACGAGCCCTCCCCTCGCGAGCCTCTG
CD2v (262-270aa)	ATCCACGAGCCCTCCCCTCGCGAGCCT
CD2v (263-271aa)	CACGAGCCCTCCCCTCGCGAGCCTCTG
CD2v (264-270aa)	GAGCCCTCCCCTCGCGAGCCT
