# Supplementary figures and images for: An Intracellular Epitope of ASFV CD2v Protein Elicits Humoral and Cellular Immune Responses
Source: Animals (Basel). 2023 Jun 12;13(12):1967. doi: 10.3390/ani13121967 (PMC10295607; doi:10.3390/ani13121967)

Figure S1

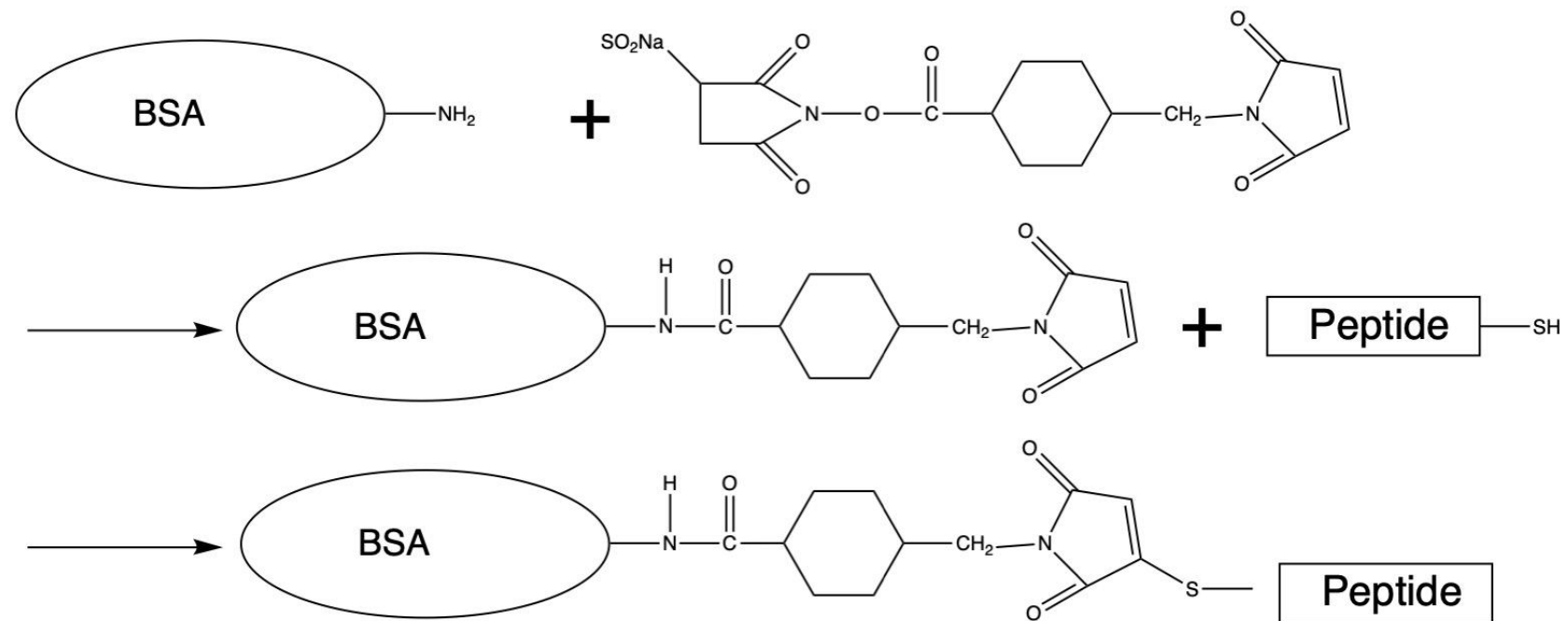

Figure S2

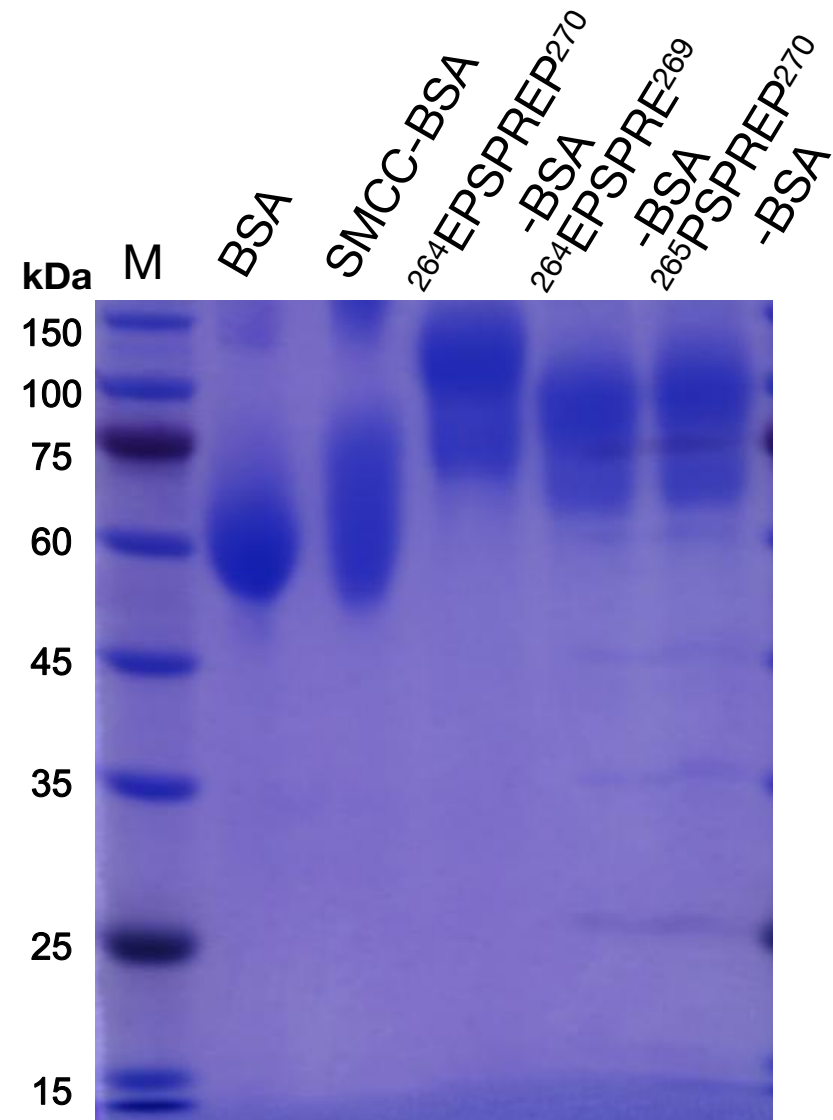

Figure S3

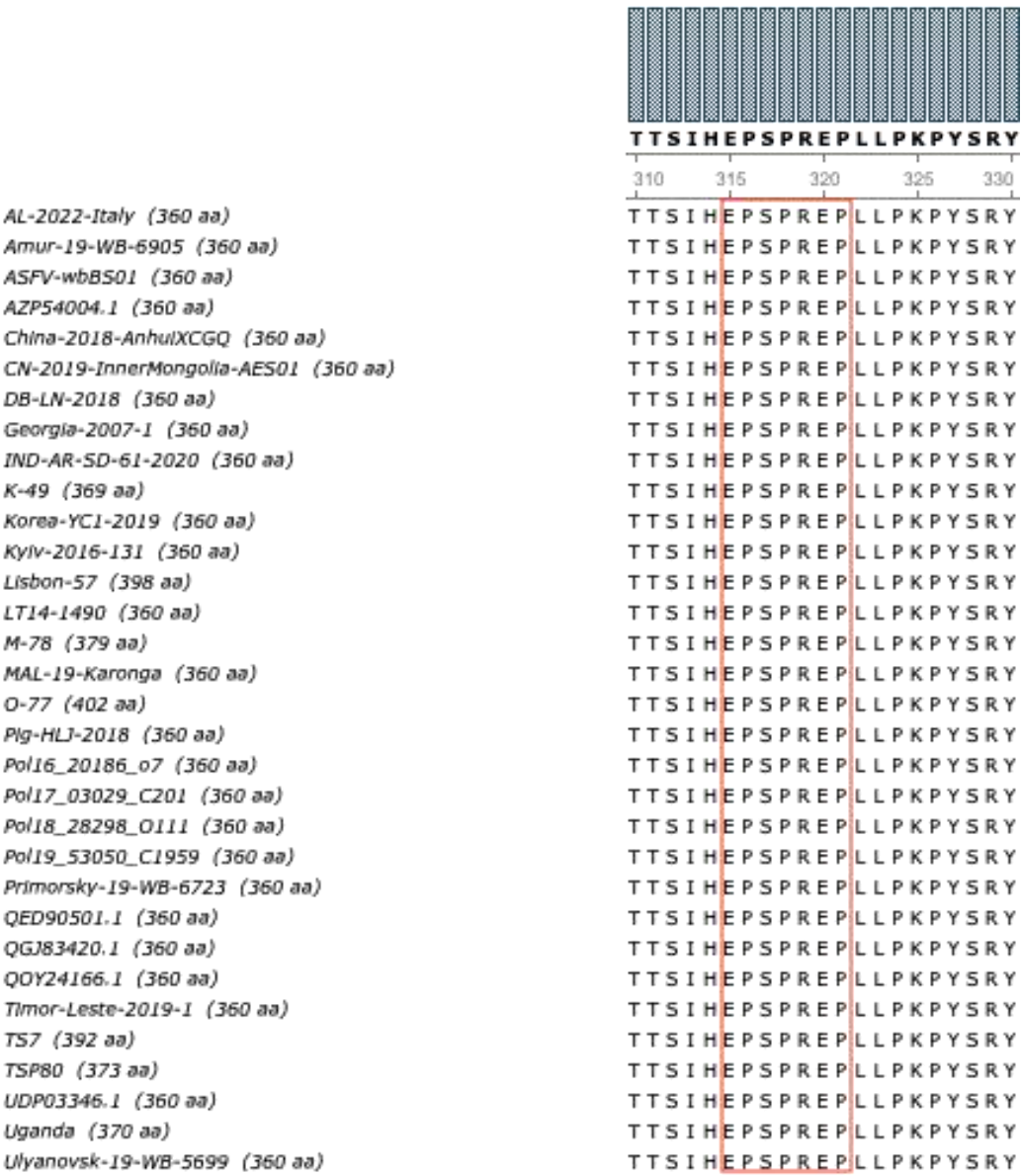

Supplement: Supplementary file 1 [file animals-13-01967-s001.zip › Supplementary figures-Revised.pdf]
